# *Cheonggukjang*, a gut microbiota-modulating Korean fermented food, improves cholesterol and bile acid metabolism

**DOI:** 10.29219/fnr.v69.13034

**Published:** 2025-10-09

**Authors:** Jung Eun Park, So-Min Oh, Jin Young Baek, Hee-Jong Yang, Do-Youn Jeong, Anna Han, Youn-Soo Cha

**Affiliations:** 1 Department of Food Science and Human Nutrition, Jeonbuk National University, Jeonju, Jeollabuk-do 54896, Republic of Korea; 2 K-Food Research Center, Jeonbuk National University, Jeonju, Jeollabuk-do 54896, Republic of Korea; 3 Microbial Institute for Fermentation Industry, Sunchang, Korea

**Keywords:** *Cheonggukjang*, hypercholesterolemia, cholesterol and bile acid metabolism, gut microbiota

## Abstract

Hypercholesterolemia (HCE) is one of the major causal factors of the development of cardiovascular disease. *Cheonggukjang* (CGJ), a representative Korean fermented soybean paste, has multiple health benefits, including cholesterol-lowering effects; however, the effects of CGJ on detailed mechanisms in cholesterol and bile acid metabolism, and the correlation between gut microbiota alterations and cholesterol and bile acid metabolism remain unclear. In this study, mice were randomly divided into the normal diet (ND, 10% fat of total kcal), high-cholesterol and fat diet (HCFD, 1% cholesterol + 45% fat of total kcal), and HCFD with 30% of two different CGJ (CGJ#1 and CGJ#2). There were no significant differences in *α*-diversity indices between the two CGJs. However, CGJ#1 was dominated by *Bacillales*, while CGJ#2 was dominated by *Bactobacillales*. Compared to HCFD, CGJ significantly reduces body weight gain, lipid accumulation in the liver and adipose tissues, and serum lipid indicators by downregulating mRNA levels involved in lipogenesis. Furthermore, CGJ strongly changes mRNA levels associated with cholesterol and bile acid metabolism in the liver and ileum and increases bile acid excretion compared with HCFD. In addition, CGJ markedly recovers HCE-derived gut microbiota dysbiosis by altering the *α*-diversity index and decreasing the *Firmicutes/Bacteroidetes* ratio. Most significantly, this recovery of HCE-derived gut microbiota composition is significantly associated with the changes of HCE-associated markers. These observations strongly suggest that CGJ, regardless of its different microbial composition, improves HCE by changing cholesterol and bile acid metabolism via reorganization of gut microbiota imbalance.

## Popular scientific summary

- *Cheonggukjang* significantly lowers body weight gain, hepatic and adipose lipid accumulation, and serum lipid levels by downregulating mRNA involved in lipogenesis and modulating cholesterol and bile acid metabolism in both the liver and the ileum.
- *Cheonggukjang* markedly improves hypercholesterolemia-induced gut microbiota imbalance by altering *α*-diversity and reducing the *Firmicutes/Bacteroidetes* ratio, with changes in microbial composition strongly correlated with improved metabolic markers.
- Both *Bacillales*-dominant and *Bactobacillales*-dominant *Cheonggukjang* exerts comparable beneficial effects on HCE, suggesting that *Cheonggukjang*’s health benefits are robust regardless of its microbial composition.

Hypercholesterolemia (HCE) is considered the primary causal factor of atherosclerosis and coronary heart disease (CHD) (1, 2). Western diet (e.g. high-fat diet, HFD) is one of the critical factors of HCE development, as it alters lipogenesis-linked enzyme expression (e.g. liver X receptor *α/β*, LXRa/*β*; acetyl-CoA carboxylase, ACC; and fatty acid synthase, FAS), consequently causing dyslipidemia (3). Previous studies have demonstrated that fermented foods elicit HCE-ameliorative effects by changing cholesterol and bile acid metabolism (4, 5). For instance, fermented black rice significantly decreases protein levels involved in cholesterol synthesis (e.g. HMG-CoA reductase, HMGCR) and storage (e.g. lecithin:cholesterol acyltransferase, LCAT) (4). Moreover, fermented black tea markedly ameliorates HCE via the alterations in gene expression associated with bile acid metabolism (e.g. cholesterol 7*α*-hydroxylase, CYP7A1; and cytochrome P450 27A1, CYP27A1) (6). These observations highlight the importance of investigating how specific fermented foods influence cholesterol and bile acid metabolism, thereby ameliorating HCE.

As a representative Korean fermented soybean paste, *Cheonggukjang* (CGJ) contains diverse microorganisms (e.g. *Bacillus subtilis*) and physiologically beneficial compounds (e.g. poly-*γ* glutamic acid, *γ*-PGA) (7, 8). Earlier studies reported diverse health-promoting effects of CGJ, including anti-atherosclerotic and anti-obesity effects (9–12). Indeed, CGJ significantly decreases body weight gain, adipose tissue weight, as well as serum levels of total cholesterol and LDL-cholesterol (9, 10); moreover, CGJ markedly downregulates adipogenesis-related mRNA expressions in the liver and adipose tissues (12). However, most of these studies mainly addressed the general outcomes, including body weight reduction, adipose tissue mass, or lipogenesis and adipogenesis-related markers, without providing mechanistic insights into cholesterol and bile acid metabolism. Furthermore, previous studies regarding the health benefits of CGJ have been mostly demonstrated by utilizing supplementation with other functional foods (e.g. red ginseng) with CGJ and/or CGJ fermented by specific strains (e.g. *Bacillus subtilis*) (13, 14); therefore, it is important to investigate the specific effects of traditional CGJ *per se*.

Gut microbiota, the community of microorganisms in the gastrointestinal (GI) tract, plays many critical roles in the maintenance of an individual’s health by regulating energy metabolism and the endocrine system (15, 16); thus, dysbiosis of gut microbiota due to an unhealthy diet is associated with the development of multiple metabolic diseases (17, 18). Recent publications have reported that diverse Korean traditional fermented foods (KTFFs) strongly restore metabolic disease-derived gut microbiota imbalance (19, 20). For example, *Gochujang* significantly ameliorates HFD-induced hepatic inflammation by recovering gut microbiota dysbiosis (19). Although it is still not fully explored, the possibilities of CGJ in the restoration of gut microbiota dysbiosis have been suggested because it has strong ameliorative effects in various colonic diseases, including inflammatory bowel disease (IBD) and constipation (21, 22); furthermore, a recent study reported that CGJ consumption was associated with modulation of the gut microbiota composition of healthy Korean subjects (23). However, the investigation regarding the mechanistic correlation between the CGJ-induced gut microbiota alterations and cholesterol and bile acid metabolism has not been fully investigated.

Thus, the goals of this study were to investigate the effects of CGJ itself in (1) cholesterol and bile acid metabolism, (2) HCE-induced dysbiosis of gut microbiota, and (3) the correlation between CGJ-derived gut microbiota flora alterations and HCE-related markers in a high-cholesterol and fat diet (HCFD)-induced hypercholesterolemic animal model.

## Materials and methods

### Material and animal studies

The CGJ was supplied by Microbial Institute for Fermentation Industry (Sunchang, Jeollabuk-do, Korea). The experimental animals were male ICR mice (4 weeks old) and purchased from DooYeol Biotech (Seoul, Korea). The mice were housed under a 12 h/12 h light/dark cycle at 23 ± 1°C and a relative humidity of 60 ± 5%. After a week of acclimation and HCFD adaptation periods, mice were randomly arranged into four groups (*n* = 10): normal diet (ND, 10% fat of total kcal), HCFD (1% cholesterol + 45% fat of total kcal), HCFD with 30% traditional CGJ (CGJ#1), and HCFD with 30% factory CGJ (CGJ#2). The 30% dose was selected based on the earlier study to ensure the physiological efficacy of CGJ (24).

The nutritional composition of the two CGJs was analyzed according to the official methods of the Korean Food Code (Ministry of Food and Drug Safety, 2021) by BioFoodLab Co., Ltd. (Seoul, Korea) (Table 1). In addition, experimental diets were provided in the form of pellets that were prepared according to the composition described in Table 2. During the experimental period, mice had free access to food and water. Food intake was recorded three times a week, and body weight (BW) was measured once a week. Energy efficiency (g/kcal) was calculated by body weight gain (g)/energy intake (kcal). After 12 weeks, serum and tissues were collected after anesthesia and stored at −80°C until analyzed. In addition, before the dissection, fecal samples were collected from individual cages for 24 h and stored at −80°C until analyzed. This study was approved by the Institutional Animal Care and Use Committee of Jeonbuk National University (NON2024-078-001).

**Table 1:** Nutritional composition of CGJ

Nutrient	CGJ#1	CGJ#2
Calorie (kcal/100 g)	211.6	179.53
Carbohydrate (g/100 g)	15.97	18.28
Dietary fiber (g/100 g)	9.12	7.91
Protein (g/100 g)	19.38	17.31
Fat (g/100 g)	7.8	4.13
NaCl (mg/100 g)	303.18	0.91

**Table 2:** The composition of the experimental diet

Content	ND	HCFD	CGJ#1	CGJ#2
Casein (g)	200	195	136.27	142.54
Methionine, DL (g)	3	3	3	3
Sucrose (g)	550	350	350	350
Lodex (g)	150	100	51.60	44.60
Starch, Corn (g)	4	50	50	50
Fiber (g)	50	50	50	50
Butter, Anhydrous (g)	0	200	176.36	187.48
Corn Oil (g)	0	10	10	10
Soybean Oil (g)	25	0	0	0
Lard (g)	20	0	0	0
Mineral (g)	50	35	35	35
Calcium Carbonate (g)	0	4	4	4
Choline Bitartrate (g)	2	2	2	2
Vitamin C (g)	1	1	1	1
Ethoxyquin (g)	0	0.04	0.04	0.04
Cholesterol, NF (g)	0	10	10	10
CGJ (g)	0	0	263.79	266.90
**Total (g)**	**1055.05**	**1010.04**	**1143.07**	**1156.57**

### Determination of microbial composition of CGJ

The microbial features of the two CGJs were determined by Next Generation Sequencing (NGS) in the Microbial Institute for Fermentation Industry (Sunchang, Jeollabuk-do, Republic of Korea). Shortly, for DNA extraction from CGJ, the DNeasy PowerFood Microbial Kit (QIAGEN, Hilden, Germany) was employed according to the manufacturer’s instructions. The 16S rRNA gene library was prepared following the 16S Metagenomic Sequencing Library Preparation protocol provided by Illumina (Illumina, San Diego, CA, USA). Sequencing was performed on the Illumina MiSeq platform using a paired-end 2 × 301 cycle configuration.

### Measurements of serum, hepatic, and fecal biochemical parameters

By using commercial kits (Asan Pharmaceutical Co., Seoul, Korea) and following the company protocol, the levels of total cholesterol (TC), triglyceride (TG), high-density lipoprotein-cholesterol (HDL), aspartate aminotransferase (AST), and alanine aminotransferase (ALT) in serum were measured. The level of very low-density lipoprotein-cholesterol (VLDL) was calculated using Friedewald’s formula (25).

For the hepatic and fecal lipid indicator analysis, the liver tissue was weighed, and feces were dried and then weighed at 0.05 g. Both samples were homogenized in a chloroform and methanol mixed solution (2:1 v/v) and centrifuged (8,000 rpm, 4°C, 15 min). The lower layer was collected and then evaporated at room temperature. The precipitate was dissolved at 5% Triton X-100 (Takara Blo Inc, Kusatsu, Shiga, Japan). The level of serum ApoB was measured based on the company’s protocol (Abcam, #ab230932).

### Fecal bile acid analysis

Fecal samples were dried for 24 h and weighed at 0.05 g. The samples were homogenized in 1 mL of cold isopropanol and incubated at 50°C to extract bile acids. After centrifugation at 10,000 g for 10 min at 4°C, the supernatant was collected and diluted 1:25 with distilled water. Bile acids were measured using the total bile acid assay kit (Cell Biolabs, STA-631).

### Histological analysis

Liver, subcutaneous fat, and epididymal fat tissues were fixed with 10% formalin for 48 h and processed further by the KP&T Company (Cheongju, Chungcheongbuk-do, Korea). Hematoxylin and eosin (H&E) staining was conducted and stained samples were analyzed by using EVOS™ M7000 Imaging System (ThermoFisher, AMF7000).

### Quantitation real-time PCR

Hepatic RNA was extracted by using the Trizol reagent (Takara Korea, Seoul, Korea). cDNA was synthesized with the PrimeScript RT Master Mix (Takara, Kyoto, Japan). Quantitation real-time PCR Reverse transcription polymerase chain reaction (qRT-PCR) analyses were performed with SYBR Green PCR Master MIX (Biosystems, Woolston, Warrington, UK) and real-time PCR (7500 Real-Time PCR System, USA). Relative quantification of gene expression was calculated relative to GAPDH, which is used as a housekeeping gene, and the relative quantification of gene expression was calculated using the 2^∆∆Ct^ method.

### Gut microbiota analysis

Fresh fecal samples (0.1 g) were collected before the experiment, stored at −80°C, and subsequently processed by the Microbial Institute for Fermentation Industry (Sunchang, Jeollabuk-do, Korea). The 16S rRNA gene library was constructed using the DNA extraction kit, and microbial composition and alpha-diversity were analyzed using the EzBioCloud 16S-based Microbiome Taxonomic Profiling software (ChunLab, Inc., Seoul, Korea).

### Statistical analysis

All results are shown as mean ± standard error of the mean (SEM). By utilizing SPSS version 23.0 (SPSS, USA), data analysis was conducted. One-way ANOVA, along with Duncan’s Multiple Range Test (a > c) was utilized and the criterion for statistical difference was set at *P* < 0.05. When further comparison between the two groups was required, the t-test was performed. *, *P* < 0.05 and **, *P* < 0.01 were considered as statistical differences.

## Results

### Distinct microbial characteristics of CGJ

**Fig. 1:**
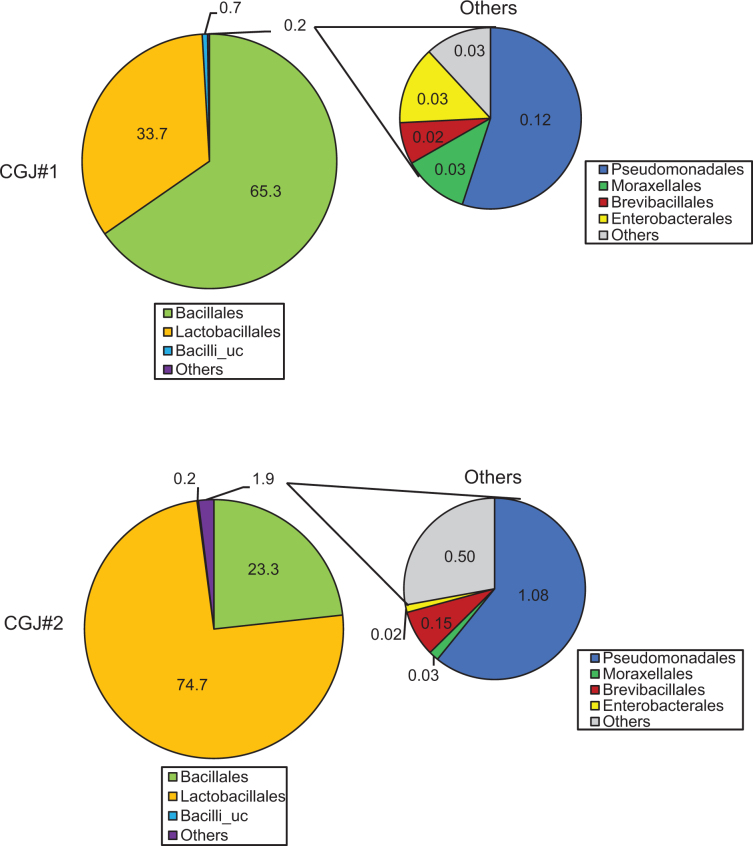
The composition of the bacterial communities of CGJs.

To analyze microbial features of two CGJs, NGS analysis was conducted. To assess the diversity and abundance of microbial composition of CGJs, *α*-diversity, including OTUs, ACEs, CHAO, Shannon, Simpson, and phylogenetic diversity of library parameters, was analyzed. Overall, the levels of diversity and abundance did not differ between the two CGJs (Table 3). Furthermore, the percentage of bacteria in both CGJs was further analyzed. The most representative order level in the total microbial community of CGJ#1 was *Bacillales* (65.3%), followed by *Lactobacillales* (33.7%) (Fig. 1, upper panel). On the other hand, CGJ#2 was dominated by *Lactobacillales* (74.7%), followed by *Bacillales* (23.3%) (Fig. 1, bottom panel). Table 4 addresses the proportion of beneficial and pernicious microbial features at the species level in the two CGJs. CGJ#1 has a relatively higher percentage of beneficial microorganisms than CGJ#2, while both CGJs do not contain pernicious microorganisms.

**Table 3:** *α*-diversity of CGJ

Sample	OTUs	ACEs	CHAO	Shannon	Simpson	Phylogenetic diversity
CGJ#1	97	209.0	171.6	1.2	0.4	206
CGJ#2	117	146.3	146.5	1.0	0.6	210

**Table 4:** The relative abundance of beneficial and pernicious bacteria in CGJs at the species level

Characteristic	Order	Species	Percentage (%)	
			CGJ#1	CGJ#2
**Beneficial**	*Bacillales*	*Bacillus subtilis*	52.61	8.89
	*Lactobaillales*	*Leuconostoc mesenteroides*	0.01	0.01
		*Weissella confusa*	0.00	0.00
		*Latilactobacillus sakei*	0.00	0.02
**Pernicious**	*Pseudomonadales*	*Acinetobacter baumannii*	0.02	0.02
	*Enterobacterales*	*Cronobacter sakazakii*	0.00	0.00

### CGJ reduces body weight gain and tissue weight in hypercholesterolemic mice

**Fig. 2:**
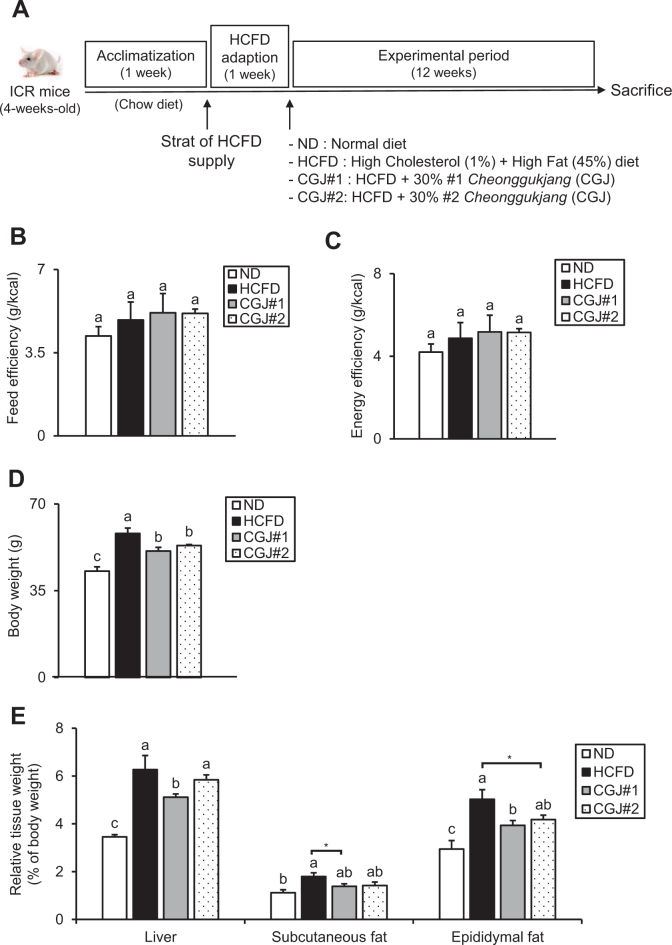
Effects of CGJ on body and tissue weights in hypercholesterolemic mice. (A) Experimental design, (B) Feed efficiency, (C) Energy efficiency, and (D) Body weight on the final day of the experimental period. (E) Relative weights of the liver, subcutaneous fat, and epididymal fat. Values are mean ± SEM. a–c means with the different letters significantly different by ANOVA with Duncan’s Multiple Range Test (*P* < 0.05, a > c). Values with an Asterisk present significant differences between the groups by unpaired t-test (*P < 0.05).

The experimental design is shown in Fig. 2A. There were no significant differences in feed efficiency and energy efficiency among the groups (Fig. 2B and C). Compared to ND, HCFD showed a significant increase in body weight on the final day, while both CGJ groups had a marked reduction in body weight compared to HCFD (Fig. 2D). HCFD had a significantly higher relative liver weight than ND, whereas both CGJ groups showed a lower relative liver weight than HCFD, with or without statistical significance (Fig. 2E, left graph). Relative to ND, the relative subcutaneous fat weight of HCFD was markedly increased. Both CGJ groups showed a decreased tendency of relative subcutaneous fat weight compared to HCFD, with or without statistical significance (Fig. 2E, middle graph). Moreover, HCFD showed strongly increased relative epididymal fat weight compared to ND, while both CGJ groups had significantly reduced relative epididymal fat weight compared with HCFD (Fig. 2E, right graph). In summary, these observations imply that CGJ markedly lowers body weight gain and adipogenesis.

### CGJ improves fat accumulation by lowering lipogenesis-related mRNA expression in hypercholesterolemic mice

With H&E staining, hepatic lipid droplets can be simply evaluated, since the fat is removed during the tissue processes, leading to the empty space and light staining (26, 27), which can be further confirmed with hepatic lipid content measurement and/or Oil-Red-O (ORO) staining. Since CGJ significantly decreases body weight gain and the relative tissue weight (Fig. 2D and E), the effects of CGJ on fat accumulation in liver and adipose tissues were evaluated via histological analysis.

**Fig. 3:**
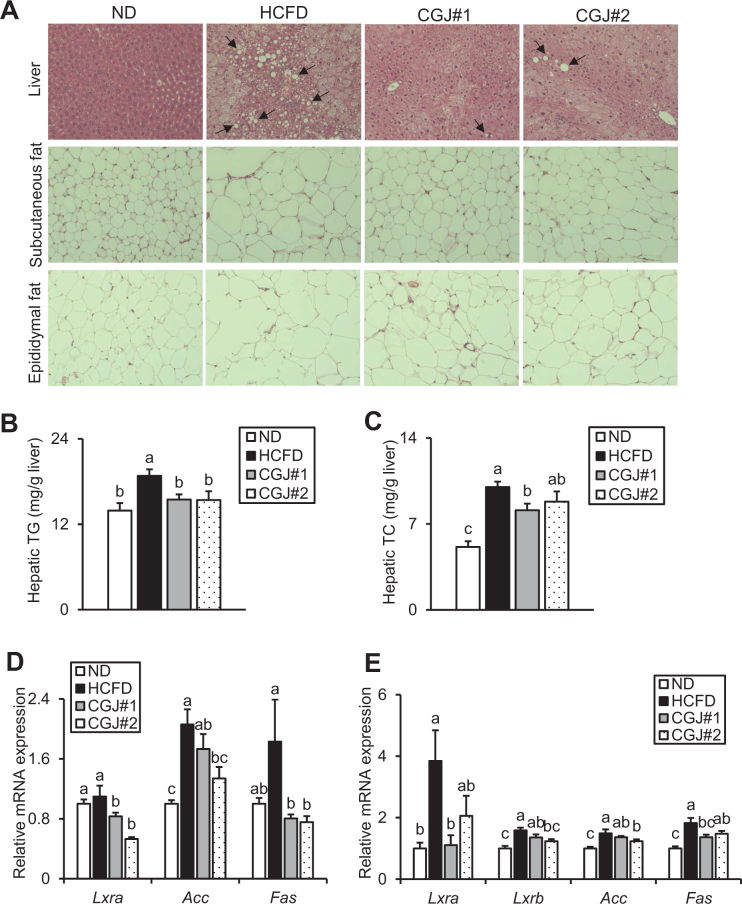
Effects of CGJ on fat accumulation and lipogenesis-related mRNA expression in the liver and adipose tissues of hypercholesterolemic mice. (A) Histological sections of the liver (arrows indicate lipid droplets), subcutaneous fat, and epididymal fat (H&E staining, magnification 200×). (B) Hepatic TG level, and (C) Hepatic TC level. (D) mRNA expression levels of lipogenesis-related genes *Lxra, Acc*, and *Fas* in the liver. (E) mRNA expression levels of lipogenesis-related genes *Lxra, Lxrb, Acc*, and *Fas* in epididymal fat. Values are mean ± SEM. a–c means with the different letters significantly different by ANOVA with Duncan’s Multiple Range Test (*P* < 0.05, a > c).

As shown in Fig. 3A, compared to ND, most hepatocytes of HCFD had a bigger fat droplet, while both CGJ groups showed a marked improvement in hepatic fat contents. In addition, in both subcutaneous fat and epididymal fat tissues, HCFD had greater adipocyte size relative to ND, whereas both CGJ groups significantly reduced adipocyte size compared to HCFD (Fig. 3A). Furthermore, the hepatic TG and TC levels of HCFD were significantly higher than ND, and both CGJ groups showed lower hepatic TG and TC levels than HCFD (Fig. 3B and C). Moreover, serum AST and ALT levels were measured to investigate the hepatic damage. Compared to ND, HCFD had markedly higher AST and ALT levels, while both CGJ groups showed significantly lower levels than HCFD (Table 5).

**Table 5:** The levels of serum parameters

Indicator	ND	HCFD	CGJ#1	CGJ#2
TG (mg/dL)	81.6 ± 5.9^ab^	87.1 ± 10.5^a^	60.6 ± 2.7^c^	66.8 ± 2.0^bc^
TC (mg/dL)	154.1 ± 5.7^c^	301.8 ± 18.5^a^	197.1 ± 12.9^b^	211.7 ± 11.6^b^
LDL (mg/dL)	71.3 ± 5.7^b^	158.6 ± 20.1^a^	98.4 ± 15.9^b^	95.5 ± 6.6^b^
HDL (mg/dL)	74.5 ± 3.3^b^	87.4 ± 10.8^ab^	88.8 ± 2.6^ab^	101.7 ± 4.7^a^
VLDL (mg/dL)	16.3 ± 1.2^ab^	17.4 ± 2.1^a^	12.1 ± 0.9^c^	13.0 ± 0.5^bc^
Apo B (ng/dL)	56.7 ± 7.9^ab^	69.1 ± 16.1^a^	30.9 ± 7.0^b^	46.8 ± 10.3^ab^
AST (IU/L)	209.4 ± 2.3^b^	223.1 ± 5.5^a^	208.1 ± 5.5^b^	205.7 ± 3.6^b^
ALT (IU/L)	100.4 ± 1.5^c^	151.1 ± 5.7^a^	120.3 ± 3.2^b^	126.3 ± 4.4^b^

Values with different superscript letters within the same column are significantly different at p < 0.05 (a>b>c).

Based on the previous observations (Fig. 3A–C), mRNA expression related to lipogenesis in the liver and epididymal fat was further investigated. The levels of hepatic *Lxra, Acc*, and *Fas* of HCFD were increased compared to ND, with or without statistical significance; however, those expressions in CGJ groups were markedly decreased compared with HCFD (Fig. 3D). Furthermore, the expressions of *Lxra, Lxrb, Acc*, and *Fas* in epididymal fat were strongly upregulated in HCFD, whereas those expressions of CGJ groups were downregulated with or without statistical significance (Fig. 3E). In short, these findings suggest that CGJ improves fat accumulation in the liver and adipose tissues by downregulating lipogenesis-associated mRNA expression, as well as ameliorating HCFD-caused liver damage.

### CGJ ameliorates HCE in hypercholesterolemic mice

To investigate the HCE-lowering effects of CGJ, the levels of serum lipid indicators were measured. Compared to ND, the serum TG level of HCFD was slightly increased, while the serum TG levels of both CGJ groups were markedly reduced relative to HCFD (Table 5). The level of serum TC was strongly elevated in HCFD relative to ND, and those indicators were significantly lowered in both CGJ groups compared to HCFD (Table 5). Furthermore, HCFD showed a significant elevation of LDL-cholesterol level relative to ND, while both CGJ groups had a significant reduction of LDL-cholesterol level compared with HCFD (Table 5). The level of VLDL in HCFD was slightly upregulated than ND, whereas those of both CGJ groups were significantly lower than HCFD (Table 5). In addition, HDL-cholesterol level of HCFD tended to elevate relative to ND, while there was no significant difference between HCFD and CGJ#1 (Table 5). The levels of HDL-cholesterol in CGJ#2 showed an increased level relative to other groups. Lastly, ApoB level in HCFD was slightly elevated compared with ND, while those indicators in both CGJ groups were markedly decreased (Table 5). These outcomes indicate that CGJ ameliorates HCE by improving serum cholesterol and lipid parameters.

### CGJ changes the cholesterol and bile acid metabolism in the liver and ileum in hypercholesterolemic mice

As CGJ markedly improves HCE (Table 5), the levels of mRNA related to cholesterol synthesis, storage, and HDL-cholesterol metabolism in the liver were investigated.

**Fig. 4:**
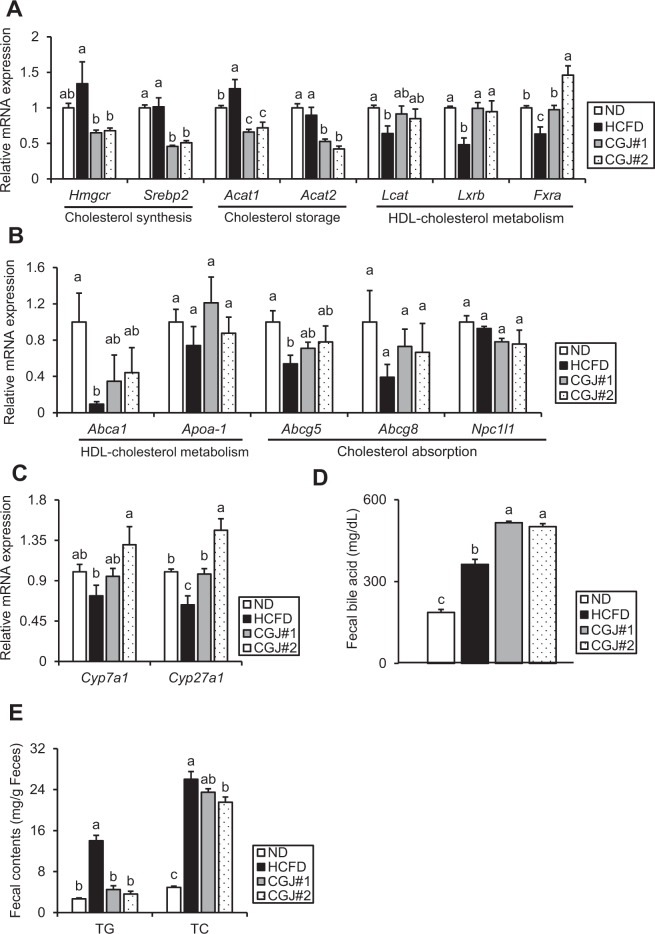
Effects of CGJ on the indicators of cholesterol and bile acid metabolism in hypercholesterolemic mice. (A) mRNA levels of cholesterol synthesis-related genes *Hmgcr* and *Srebp2*, cholesterol storage-related genes *Acat1* and *Acat2*, and HDL-cholesterol metabolism-related genes *Lcat, Lxrb*, and *Fxra* in the liver. (B) mRNA levels of HDL-cholesterol metabolism-related genes *Abca1* and *Apoa-1*, and cholesterol absorption-related genes *Abcg5, Abcg8*, and *Npc1l1* in the ileum. (C) mRNA levels of bile acid synthesis-related genes *Cyp7a1* and *Cyp27a1* in the liver. (D) Bile acid levels in feces. (E) Triglyceride (TG) and total cholesterol (TC) levels in feces. Values are presented as mean ± SEM. Different letters (a–c) indicate significant differences determined by one-way ANOVA followed by Duncan’s Multiple Range Test (*P* < 0.05, a > c).

Compared to ND, HCFD showed a slight increase of hepatic mRNA expressions related to cholesterol synthesis, including *Hmgcr* and sterol regulatory element-binding protein 2 (*Srebp2*), whereas both CGJ groups had a significant reduction of those mRNA expressions compared to HCFD (Fig. 4A). Moreover, HCFD had a significant upregulation of hepatic acyl-CoA:cholesterol acyltransferases 1 (*Acat1*) level compared with ND, but not *Acat2* expression; however, both CGJ groups had a marked reduction of hepatic Acat1 and Acat2 mRNA expression relative to HCFD (Fig. 4A). Furthermore, hepatic mRNA levels associated with HDL-cholesterol metabolism, such as *Lcat, Lxrb*, and farnesoid X receptor (*Fxra*) in HCFD were significantly lower than ND, whereas both CGJ groups showed a strong upregulation of those mRNA levels relative to HCFD (Fig. 4A).

Furthermore, mRNA expression associated with HDL-cholesterol metabolism and cholesterol absorption in the ileum was also evaluated because the ileum is the main site for cholesterol metabolism, including nascent HDL formation and cholesterol absorption (28). Compared with ND, the mRNA level of ATP-binding cassette transporter (*Abca1*) in HCFD was significantly reduced, while both CGJ groups showed an increased tendency (Fig. 4B). Although there were no significant differences in the expression of apolipoprotein A1 (*Apoa-1*) among all groups, HCFD showed a reduced trend of Apoa-1 mRNA expression relative to ND, whereas both CGJ groups showed an elevated tendency (Fig. 4B). Moreover, the mRNA levels of ATP-binding cassette subfamily G member 5 (*Abcg5*) and *Abcg8* in HCFD were downregulated with or without statistical significance compared to ND, but the expression of those enzymes in CGJ groups was upregulated with or without statistical significance relative to HCFD (Fig. 4B). Even though there was no marked difference in the mRNA expression of Niemann-pick C1-like 1 (*Npc1l1*) among all groups, but CGJ group showed a lower tendency relative to ND and HCFD (Fig. 4B).

Next, the effects of CGJ on bile acid metabolism were further investigated, as it shows the significant effects of CGJ on gene expression in the liver and ileum (Fig. 4A, B). The hepatic *Cyp7a1* mRNA level of HCFD was slightly decreased compared with ND, but its expression in both CGJ groups was strongly increased relative to HCFD (Fig. 4C); moreover, HCFD showed a markedly lower hepatic *Cyp27a1* level than ND, whereas CGJ groups had significantly higher levels of *Cyp27a1* mRNA expression than HCFD (Fig. 4C). In addition, compared to ND, HCFD had a significantly higher bile acid content in feces, but CGJ showed a further increase in fecal bile acid concentration compared to HCFD (Fig. 4D).

The elevation of fecal fat excretion represents dysregulation of lipid metabolism (29); therefore, fecal TG and TC contents were measured. While HCFD showed significantly increased fecal TG and TC levels, both CGJ groups had a marked reduction of TG and TC contents in feces (Fig. 4E). To sum up, these outcomes suggest that CGJ changes cholesterol and bile acid metabolism in both the liver and the ileum by altering related gene expressions.

### CGJ recovers gut microbiota diversity and composition in hypercholesterolemic mice

To understand the gut microbiota imbalance in HCFD-induced hypercholesterolemic mice and the direct effects of CGJ on it, gut microbiota dysbiosis via *α*-diversity analysis and detailed changes in gut microbiota composition at the phylum and genus levels were analyzed.

**Fig. 5:**
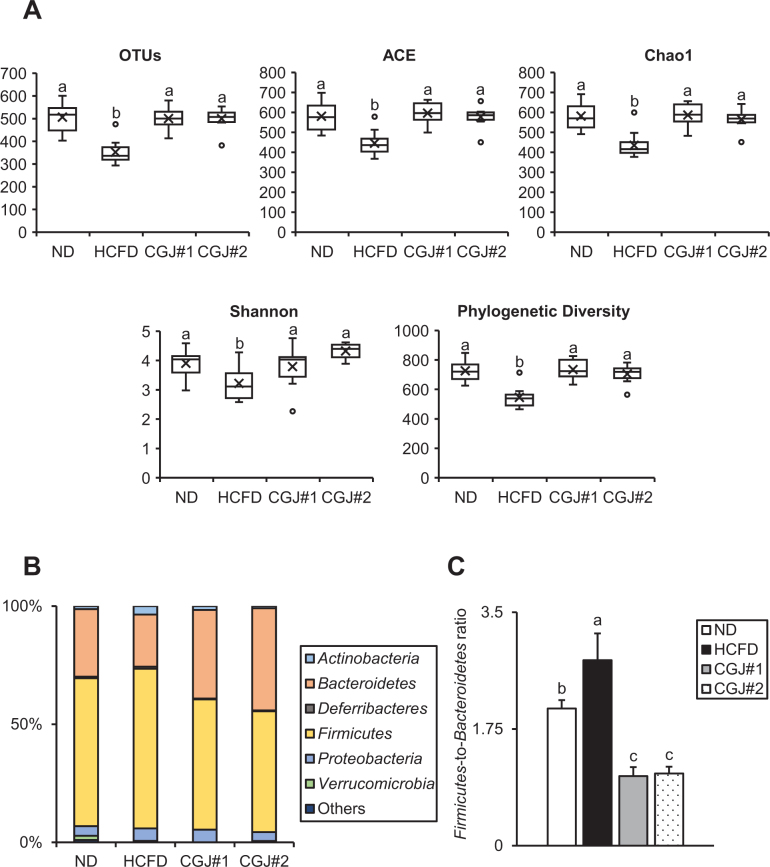
Effects of CGJ on gut microbiota diversity and composition in hypercholesterolemic mice. (A) Operational Taxonomic Units (OTUs), ACE, Chao1, Shannon index, and Phylogenetic Diversity at the species level. (B) Relative abundance of microbial communities at the phylum level in mice. (C) *Firmicutes*-to-*Bacteroidetes* (F/B) ratio in mice. Values are presented as mean ± SEM. Different letters (a–c) indicate significant differences determined by one-way ANOVA followed by Duncan’s Multiple Range Test (*P* < 0.05, a > c).

The results of *α*-diversity analysis, including Operational Taxonomic Units (OTUs), ACE, Chao1, Shannon index, and Phylogenetic diversity, suggested that HCFD has decreased microbial species richness compared to ND, while both CGJ groups show the recovery of this distribution and diversity (Fig. 5A). In all groups, *Firmicutes* and *Bacteroidetes* were dominant species at the phylum level (Fig. 5B). Compared to ND, HCFD showed reduced levels of *Bacteroidetes* and elevated levels of *Firmicutes*, while both CGJ groups significantly restored these alterations compared with HCFD (Fig. 5B). Thus, the *Firmicutes*-to-*Bacteroidetes* ratio (F/B ratio) was significantly increased in HCFD compared with ND, while both CGJ groups showed markedly decreased F/B ratio compared with HCFD (Fig. 5C).

**Fig. 6:**
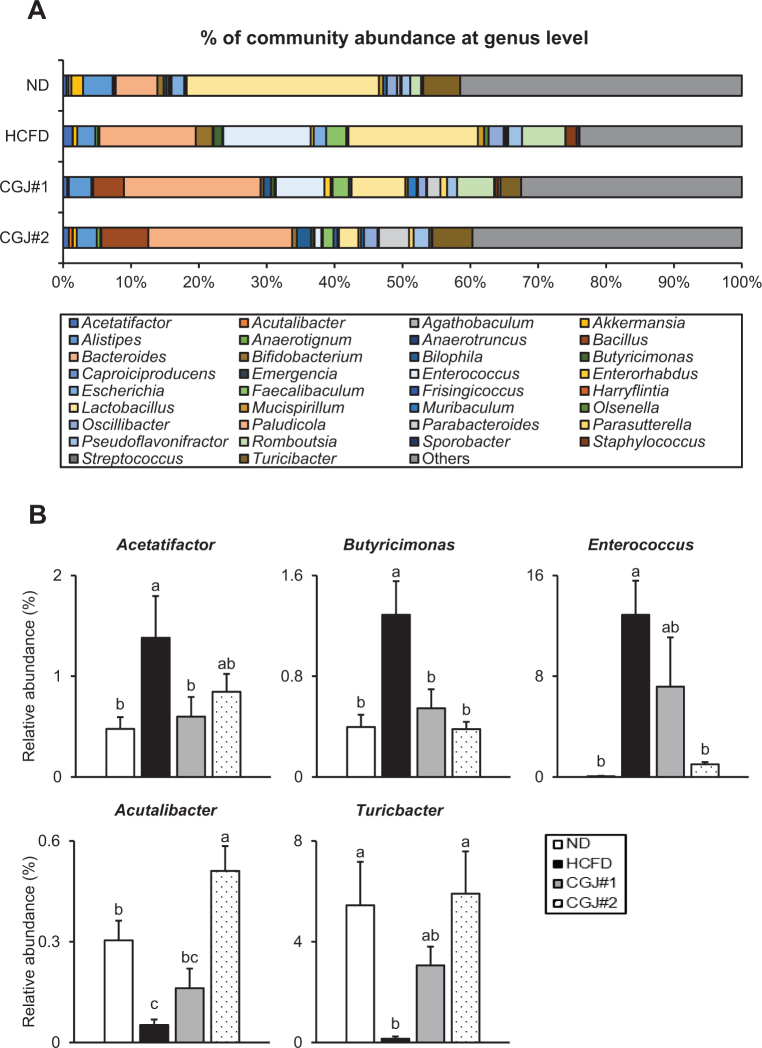
Effects of CGJ on gut microbiota diversity and composition in hypercholesterolemic mice. (A) Microbial community composition at the genus level in mice. (B) Relative abundance of specific genera, including *Acetatifactor, Butyricimonas, Enterococcus, Acutalibacter*, and *Turicibacter*. Values are presented as mean ± SEM. Different letters (a–c) indicate significant differences determined by one-way ANOVA followed by Duncan’s Multiple Range Test (*P* < 0.05, a > c).

At the genus level, gut microbiota composition was dramatically changed based on the groups (Fig. 6A). Specifically, compared to ND, the levels of *Acetatifactor, Butyricimonas*, and *Enterococcus* were significantly elevated in HCFD, whereas those of both CGJ groups were significantly decreased compared with HCFD (Fig. 6B, upper panel). On the other hand, the levels of *Acutalibacter* and *Turicbacter* in HCFD were markedly decreased compared with ND. At the same time, both CGJ groups showed strong elevations of the levels of those microbiotas (Fig. 6B, bottom panel). These observations imply that CGJ strongly restores gut microbiota imbalance in hypercholesterolemic mice.

### Correlation between HCE indices and major gut microbiota

**Fig. 7:**
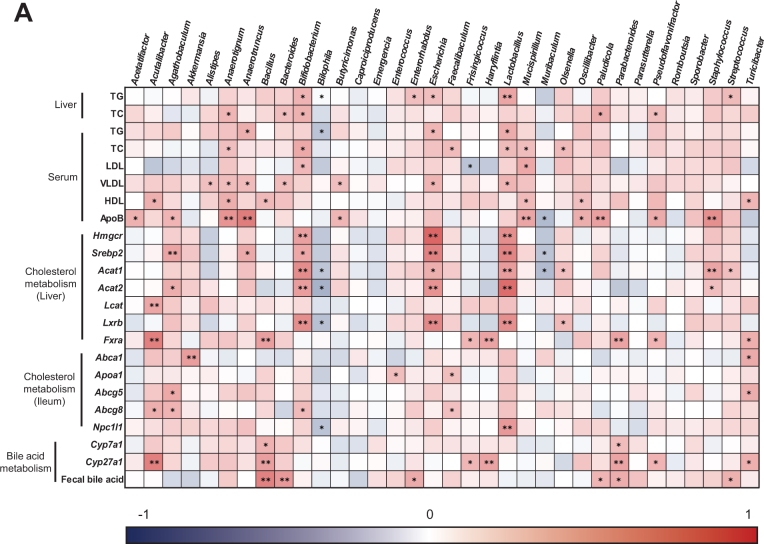
Effects of CGJ on Spearman correlation between major gut microbiota and hepatic inflammation-related parameters in hypercholesterolemic mice. The color intensity indicates the strength of the correlation (dark blue: negative correlation, dark red: positive correlation). * *P* < 0.05, ** *P* < 0.01, *** *P* < 0.001.

Spearman’s correlation analysis was conducted to understand the correlation between the index of cholesterol and bile acid metabolism in HCFD-induced hypercholesterolemic mice and gut microbiota composition at the genus level (Fig. 7). *Anaerotignum, Bifidobacterium, Escherichia, Lactobacillus, Mucispirillum*, and *Turicibacter* were positively associated with serum and hepatic lipid indicators. Moreover, the mRNA expressions involved in hepatic cholesterol metabolism (e.g. *Hmgcr, Sreba2, Acat1*, and *Acat2*) were positively associated with *Acutalibacter, Agathobaculum, Bifidobacterium*, Escherichia, and *Lactobacillus*, whereas those indicators were negatively correlated with *Bilophila* and *Muribaculum*. In addition, *Acutalibacter, Bacillus, Frisingicoccus, Harryflintia, Parabacterioides, Pseudoflavonifracter*, and *Turicibacter* were positively associated with hepatic *Cyp27a1* mRNA expression. Furthermore, *Bacillus, Bacteroides, Enterorhabdus, Paludicola, Parabacteroides*, and *Streptococcus* were directly associated with fecal bile acid. These findings indicate that gut microbiota community alterations are markedly correlated with HCE and its amelioration by CGJ.

## Discussion

Multiple health benefits of CGJ have been demonstrated, including cholesterol-lowering effects (9–11); however, the precise underlying mechanisms of its effects in cholesterol and bile acid metabolism have not been fully scrutinized yet. Moreover, the direct involvement of CGJ in the gut microbiota community and its derived health-beneficial outcomes in cholesterol and bile acid metabolism has also not been investigated. This study reported that CGJ significantly 1) ameliorates HCE indicators, including fat accumulation in liver and adipose tissues, as well as the levels of serum lipid parameter, 2) alters cholesterol and bile acid metabolism in the liver and ileum, and 3) recovers gut microbiota dysbiosis, which was associated with the parameters related to cholesterol and bile acid metabolism in HCFD-induced hypercholesterolemic mice.

The cholesterol-lowering effects of CGJ have been well-known (9, 14, 30). Moreover, CGJ also has anti-obesity effects by reducing body weight gain, epididymal fat weight, and hepatic lipid levels (9, 10, 24). This study also observed that CGJ markedly decreases body weight gain, adipogenesis, and lipid accumulation in the liver and adipose tissues by downregulating lipogenesis-related mRNA expressions. In addition, CGJ also markedly improves HCE by reducing serum lipid indicator levels, including TG, TC, and lipoproteins (e.g. VLDL and ApoB). An earlier study observed that CGJ dramatically improves TC, VLDL, LDL, and atherogenic index in female rats (30). After menopause, the risk of HCE in women is strongly increased as LDL-cholesterol levels increase up to 25%, subsequently, elevating the risk of arteriosclerosis (31, 32). Therefore, it will be interesting to study the HCE-lowering effects of CGJ specifically in a postmenopausal study design by utilizing female mice to evaluate the gender-specific potential of CGJ. Moreover, investigation regarding the effects of CGJ on muscle and bone weights will also be interesting to establish unexplored CGJ’s roles in muscle and bone metabolism. The improvements in cholesterol and bile acid metabolism are strongly related to HCE amelioration (4–6). This study found that CGJ significantly changes the mRNA levels involved in cholesterol metabolism, including synthesis (*Hmgcr* and *Srebp2*), storage (*Acat1* and *Acat2*), absorption (*Abcg5, Abcg8*, and *Npc1l1*), and HDL-cholesterol (*Lcat, Lxrb, Fxra, Abca1*, and *Apoa-1*) in the liver and ileum. Furthermore, CGJ alters the gene expressions related to bile acid synthesis (*Cyp7a1* and *Cyp27a1*) as well as increases fecal bile acid excretion. *Bacillus subtilis*, the richest bacteria in CGJ, generates diverse proteolytic enzymes, producing distinct amino acids, such as *γ*-PGA, improving digestibility, and elevating nutrient levels (e.g. calcium and riboflavin) (7, 8). *γ*-PGA has been considered one of the major functional compounds exerting diverse health-beneficial effects of soybean-based foods (33, 34); however, its direct involvement in the health advantageous functions of KTFFs, including CGJ, has not been fully studied yet. Moreover, the potential bioactive compounds in CGJ that elicit its beneficial outcomes have not been completely investigated. Therefore, future research will be mandatory to study unknown bioactive compounds in CGJ and their detailed roles in CGJ’s health-beneficial outcomes, including *γ*-PGA.

The potential functions of CGJ in gut microbiota composition have been implied because of its effects in the improvement of colonic diseases (21, 22); furthermore, clinical outcomes have reported that CGJ changes gut microbiota composition (23, 35). This study found that CGJ dramatically restores gut microbiota dysbiosis in HCFD-induced hypercholesterolemic mice, leading to an increase in the diversity of the gut microbiota community and a decrease in the F/B ratio. According to previous studies, fermented soybean pastes showed significantly stronger health-beneficial effects relative to non-fermented soybeans (36–38). For example, CGJ increases overall immunity and immune safety relative to an unfermented raw soybean mixture (38). Although this study showed the significant role of CGJ on gut microbiota, future investigation is required to compare these effects with a non-fermented soybean control group. Moreover, it will be pivotal to investigate shorter and/or longer effects of CGJ on the gut microbiota in aspects of HCE-lowering effects because fermented foods can affect the gut microbiota in both the short and long term (39).

Importantly, this study not only reported that the recovery of gut microbiota imbalance by CGJ is strongly correlated with HCE-associated parameters, including serum lipid levels, mRNA expression related to cholesterol and bile acid metabolism, but also fecal bile acid excretion. Interestingly, CGJ markedly reduces short-chain fatty acids (SCFAs)-producing bacterial species, including *Acetatifactor* and *Butyricimonas* (40), suggesting the possibility of changes in gut microbiota-derived SCFAs production. According to the previous study, the fecal SCFAs profile was correlated with the improvement of serum lipid parameters in HCE (41), implying the potential functions of gut microbiota-derived SCFAs in cholesterol and bile acid metabolism to ameliorate HCE. Therefore, the effects of CGJ on SCFA production and its detailed functions in CGJ’s HCE-ameliorative effects should be studied to solidify the connection among CGJ, gut microbiota, and cholesterol-lowering effects.

In conclusion, this study reports that CGJ lowers HCE by integrating its effects in adipogenesis, lipid accumulation, serum lipid levels, mRNA expression involved in cholesterol and bile acid metabolism, and gut microbiota imbalances. Furthermore, this study is the first to demonstrate the correlation between CGJ’s anti-hypercholesterolemic effects and the recovery in gut microbiota imbalance. In the future, ORO staining of liver tissue and a quantitative evaluation of hepatocyte morphology or vacuolation will be valuable to understand the detailed roles of GCJ in hepatic lipid metabolism as well as phenotypes, since the liver is one of the major organs for cholesterol and bile acid metabolism. These findings provide a concrete scientific basis for understanding the strong roles of CGJ in the gut microbiota and HCE. Moreover, this study also provides the potential of CGJ as a functional dietary intervention to control cholesterol-related metabolic diseases.

## Data Availability

All of the data are available with a reasonable request from the corresponding author.
